# Biomarker Signature in Aqueous Humor Mirrors Lens Epithelial Cell Activation: New Biomolecular Aspects from Cataractogenic Myopia

**DOI:** 10.3390/biom13091328

**Published:** 2023-08-29

**Authors:** Maria De Piano, Andrea Cacciamani, Bijorn Omar Balzamino, Fabio Scarinci, Pamela Cosimi, Concetta Cafiero, Guido Ripandelli, Alessandra Micera

**Affiliations:** 1 Research and Development Laboratory for Biochemical, Molecular and Cellular Applications in Ophthalmological Science, IRCCS-Fondazione Bietti, 00184 Rome, Italy; maria.depiano@fondazionebietti.it (M.D.P.); bijorn.balzamino@fondazionebietti.it (B.O.B.); 2 Surgical Retina Research Unit, IRCCS-Fondazione Bietti, 00184 Rome, Italy; andrea.cacciamani@fondazionebietti.it (A.C.); fabio.scarinci@fondazionebietti.it (F.S.); pamela.cosimi@fondazionebietti.it (P.C.); guido.ripandelli@fondazionebietti.it (G.R.); 3 Anatomic Pathology Unit, Fabrizio Spaziani Hospital, 03100 Frosinone, Italy; concettacafiero@gmail.com

**Keywords:** myopia, cataract, aqueous, lens epithelial cells, inflammation, ETM, biomarkers

## Abstract

Inflammatory, vasculogenic, and profibrogenic factors have been previously reported in vitreous (VH) and aqueous (AH) humors in myopic patients who underwent cataract surgery. In light of this, we selected some mediators for AH and anterior-capsule-bearing lens epithelial cell (AC/LEC) analysis, and AH expression was correlated with LEC activation (epithelial–mesenchymal transition and EMT differentiation) and axial length (AL) elongation. In this study, AH (97; 41M/56F) and AC/LEC samples (78; 35M/43F) were collected from 102 patients who underwent surgery, and biosamples were grouped according to AL elongation. Biomolecular analyses were carried out for AH and LECs, while microscopical analyses were restricted to whole flattened AC/LECs. The results showed increased levels of interleukin (IL)-6, IL-8, and angiopoietin-2 (ANG)-2 and decreased levels of vascular endothelium growth factor (VEGF)-A were detected in AH depending on AL elongation. LECs showed EMT differentiation as confirmed by the expression of smooth muscle actin (α-SMA) and transforming growth factor (TGF)-βR1/TGFβ isoforms. A differential expression of IL-6R/IL-6, IL-8R/IL-8, and VEGF-R1/VEGF was observed in the LECs, and this expression correlated with AL elongation. The higher VEGF-A and lower VEGF-D transcript expressions were detected in highly myopic LECs, while no significant changes were monitored for VEGF-R transcripts. In conclusion, these findings provide a strong link between the AH protein signature and the EMT phenotype. Furthermore, the low VEGF-A/ANG-2 and the high VEGF-A/VEGF-D ratios in myopic AH might suggest a specific inflammatory and profibrogenic pattern in high myopia. The highly myopic AH profile might be a potential candidate for rating anterior chamber inflammation and predicting retinal distress at the time of cataract surgery.

## 1. Introduction

Myopia is the most common worldwide public health refractive error in modern societies, causing vision reduction or even severe visual loss when associated with pathological retinal changes [1,2]. Genetic, epigenetic, and environmental factors influence the development of myopia and/or exacerbate the disease progression, or even trigger the involvement of vitreoretinal compartments [3,4,5]. The main indicator of the development of myopia is axial length (AL) elongation, which represents a major factor leading to visual impairments. No treatments are available to reverse the disease or to counteract AL elongation if progressive. AL elongation has been associated with morphological and functional changes in the cellular part of crystalline (lens epithelial cells, LECs) long-standing active extracellular matrix (ECM) turnover, and retinal microvasculature density in the macula [6,7,8]. Changes in aqueous humor (AH) composition could mirror LEC activation and myopia progression and in turn influence the LECs’ activity (ion-transport/metabolites imbalance, oxidative stress, cell apoptosis, and abnormal growth of fiber cells) [9,10]. The transforming growth factor (β) was the first profibrogenic factor to be investigated for LEC activation in myopia studies [11,12]. TGFβ expression can explain the epithelial to mesenchymal transition (EMT) and the expression of α-smooth muscle actin (α-SMA) as well as the scleral ECM makeover observed in LEC from myopic eyes [13,14,15,16]. Local (AH) and systemic (plasma) associations between the increased levels of TGF-βs, matrix metalloproteinase (MMP), and tissue inhibitors of MMP (TIMP) have been observed in myopic patients [15,17,18,19]. Other studies have confirmed TGFβ involvement in AH from high myopic patients and PEDF involvement in intraocular CNV lesions, but circulating TGFβ and PEDF had no significant correlation with high myopia or intraocular CNV disease [20]. Transcriptomic and proteomic studies have confirmed the presence of inflammatory and profibrogenic markers in high myopia and have identified some of the angiogenic/angiostatic factors linked to myopia progression and macular involvement, such as VEGF [21,22]. In addition to the consistent literature of the last few years, several questions on the mechanisms underlying the development of myopia and progression remain unanswered. An interesting study highlighted the presence of some inflammatory mediators, precisely, IL-6, IL-8, VEGF-A, and ANG-2, in the AH and VH of myopic subjects, suggesting a link between AH mediators and retinal involvement [23,24,25,26].

Our study aimed at i. confirming the presence of IL-6, IL-8, VEGF-A, and ANG-2 in myopic AH, ii. addressing the question as to whether LECs might contribute to and/or respond to this AH protein print, and iii. evaluating the association between an AH protein signature and the markers of LEC activation and AL elongation. Comparisons were carried out between myopia and high myopia using biostrumental (AL elongation) and biomolecular (α-SMA expression) parameters.

## 2. Materials and Methods

This observational single-point study (June 2020 to June 2021) was approved by the intramural ethical committee (IFO/Bietti, Rome, Italy, number 99/20/FB). The study was conducted in accordance with the ethical standards stated in the Declaration of Helsinki. Written informed consent was obtained from patients before enrollment in the study and before clinical and biostrumental data collection and biosampling.

### 2.1. Study Population: Clinical Assessment and Sampling

A total of 102 patients (44M/58F) elective for cataract surgery participated in this study. The main demographic data and AL elongation values are summarized according to the three main subgroups of the study in Table 1.

Patients were recruited according to inclusion and exclusion criteria. Essential exclusion criteria included previous intraocular surgery, other ocular diseases, the presence of hypermature cataracts, and the presence of topical therapy (glaucoma). Indeed, patients with systemic autoimmune (Sjögren’s syndrome), endocrine (thyroiditis), and metabolic (diabetes) diseases were excluded from the study.

Patients underwent pre-surgical visits, including anamnesis, registration of comorbidities, and a full ophthalmological examination comprising optometric tests (AL measurement) and slit-lamp inspection with complete pupillary dilation. Only 97 out of 102 patients undergoing phacoemulsification adhered to sample collection. Briefly, 97 (41M/56F) AH samples were processed for protein assays (Ella™ and ELISA) while 78 (35M/43F) AC/ LECs samples were used for microscopical analysis and molecular evaluations. Five (5) samples were excluded because they belonged to patients who dropped out of the study. Samples were grouped as emmetropia or controls (≤24 mm AL), myopia (24–26 mm AL), and high myopia (≥26 mm AL).

### 2.2. Pre-Analytical Procedures

AH (10–200 µL) fluids were sampled at the beginning of surgery and immediately after incision, and AC specimens were collected during capsulorhexis. AH samples were quickly centrifuged (1300 rpm/7 min), and cell-free supernatants were collected and supplemented with protease inhibitors (Pierce; Thermo Fisher Scientific, Milan, Italy). Samples were spectrophotometrically analyzed (SPECTROstar Nano reader, BMG LABTECH, Ortenberg, Germany) and stored at −20 °C.

Unfixed AC specimens were devoted to LEC analysis according to a standard procedure, while prefixed AC specimens (ThinPrep™ Cyt Solution; Cytyc Corp, Marlborough, MA, USA) were processed for microscopical analysis.

### 2.3. Analytical Procedures

The biomarker panel included the following proteins and specific receptors: IL-6, IL-8, VEGF-A, ANG-2, TGFβs, and α-SMA.

#### 2.3.1. Ella™ and ELISA Analysis

The multiplex Ella™ platform was used to quantify the levels of IL-6, IL-8, and ANG-2 in AH samples (20 emmetropia; 15 myopia; 13 high myopia). Briefly, 10 μL of clear samples was 1:3 diluted and added to cartridges, according to a standard procedure provided by the manufacturers (Protein Simple, San Jose, CA, USA). All steps in the immunoassay procedure and data acquisition were carried out automatically. Cartridges included built-in standard curves, and samples were run as internal triplicates. Single data (pg/mL) for each sample were automatically calculated and provided in “xls format” for statistical analysis.

A commercial ELISA test was used for quantifying the VEGF-A protein in AH samples (21 emmetropia; 15 myopia; 13 high myopia). Samples were diluted with an extraction buffer and loaded on 96-well pre-coated plates (EH2VEGF-A; Thermo Scientific, Waltham, MA, USA). The standard curve range and limit of detection were, respectively, 63–4000 pg/mL and 2.5 pg/mL. Absorbance (OD) values were recorded after reading the plates at λ450 nm (corrected to λ570 nm) in a 96-well plate reader platform (Sunrise; Tecan Group Ltd., Männedorf, Switzerland).

#### 2.3.2. Molecular Analysis: 2-Step Real-Time RT-PCR Analysis

Forty-eight AC/LEC samples were used for molecular analyses (20 emmetropia, 15 myopia, and 13 high myopia). Total RNA was extracted in Trifast reagent solution (1:1; EuroClone, Milan, Italy), and normalized RNA samples (100 ng/sample) were used to synthesize cDNA (LifePRO/BIOER, Euroclone, Milan, Italy) using the IMPROM kit and random primers (Promega, Madison, WI, USA). Specific amplifications were carried out using one-intron spanning pair primers that were properly designed for the following target genes, as well as for α-SMA and actin: IL-6/IL-6R, IL-8/IL-8R, VEGFs/VEGF-Rs, and TGFβs/TGFβR1. Information on primer sequences (targets and housekeeping), annealing temperature, and amplicons’ length is reported in Supplementary Table S1.

Normalized cDNAs were amplified by using an SYBR green hot-start PCR master mix (Hydra Taq; Biolab, Biocell, Rome, Italy) in a 48-well microplate real-time PCR platform (Eco™ Illumina, San Diego, CA, USA). Specific amplifications were tested by verifying the single curve specific for each amplicon. The amplification protocol included the following: pre-hold (5 min at 50 °C) and pre-incubation for 15 min at 95 °C. Each of the 39 amplification cycles consisted of 30 s/94 °C denaturation, followed by a specific annealing step at 58–60 °C and 30 s/72 °C extension. Annealing was set at appropriate temperatures Ta = (Tm–5 °C) and verified for specificity via grading. The melting curve was registered from 56.0 °C to 94.1 °C (0.3 °C hold for 00:00:01 between reads). Single melting curves were verified at the end of each amplification, cycle threshold (Ct) values were detected, and target gene expressions were provided by software (row data) according to the 2-(ΔΔCt) formula (ΔΔCt = ΔCt sample − ΔCt calibrator). The single-target gene expressions (fold changes, FC) were expressed in the log2 scale, as directly provided by Illumina software with respect to the emmetropic group (normal values). REST 384–2006 software was also used to estimate changes in transcripts’ expression, as calculated with respect to two referring genes (H3 and GAPDH).

#### 2.3.3. Microscopy, Digital Imaging, and Densitometry

PAS staining was carried out on whole flattened AC/LECs (30 specimens; 10 samples/subgroup) according to the standard procedure combined with nuclear Hematoxylin staining (Bio-Optica srl, Milan, Italy). Light microscopy images were acquired by using the direct microscope (T200; Nikon, Tokyo, Japan) connected to a digital camera and open-source Zen 2 (blue edition) (Zeiss Imaging Software, Milan, Italy). Double immunofluorescent analysis was also performed. After a brief permeabilization and autofluorescence reduction treatment, whole flattened AC/LECs were probed using primary antibodies that are specific for human anti-smooth muscle actin (α-SMA) monoclonal (mouse) and actin polyclonal (goat) antibodies (4 μg/mL; Sigma-Aldrich, Milan, Italy). Specific binding was detected using Cy-2-conjugated secondary anti-mouse antibody (1:300; Jackson ImmunoResearch, West Grove, PA, USA). Nuclear staining was performed using DAPI (5 μg/mL; Molecular Probes, Invitrogen, Milan, Italy) diluted in PBS supplemented with RNase (20 μg/mL; Molecular Probes). Irrelevant isotype-matched IgG antibodies (Vector Labs. Ltd., Burlingame, CA, USA) were incubated in parallel and used for background subtraction purposes (internal controls). Images were acquired (×20/0.50 Plan Fluor and ×40/0.75 Plan Fluor objectives) by using a direct epifluorescent microscope (Ni Eclipse) equipped with a DS-Ri digital camera and NIS Elements Imaging Software (Nikon).

### 2.4. Statistical Analysis

All efforts were devoted to quickly freezing the tissues, and steps in the sampling procedure were standardised to avoid the surgery’s influence on the results of real-time PCR. As shown in Table 1, the study population comprised individuals whose age, sex, and comorbidities matched those of the inclusion and exclusion criteria. Specimens were clustered according to AL elongation ranges (emmetropia (≤24 mm AL), myopia (24–26 mm AL), and high myopia (≥26 mm AL)). The distribution of quantitative row data (mean ± SEM; pg/mg total proteins) for biochemical analysis or molecular ones (2log expression) was analyzed using Shapiro–Wilk tests, confirming the assumption of records coming from a normally distributed population (Prism vs. 10.0.0; GraphPad Software Inc., San Diego, CA, USA). The Levine test was also carried out to verify the assumption of equal variance between subgroups. The Pearson test was used to correlate protein data with AL elongation, while REST-ANOVA-coupled analysis was carried out to identify significant changes in transcript expression. Significant levels are indicated in the panels with asterisks (*p* value summary: ns, not significant; * *p* ≤ 0.05; ** *p* ≤ 0.005; *** *p* ≤ 0.0005; **** *p* ≤ 0.0001).

## 3. Results

In this study, all subjects that were elective for cataract surgery were recruited according to the inclusion/exclusion criteria. No association between gender and myopia was observed, as shown in Table 1 (*p* > 0.05).

### 3.1. AH Expression of IL-6, IL-8, VEGF-A, and ANG-2

**Figure 1:**
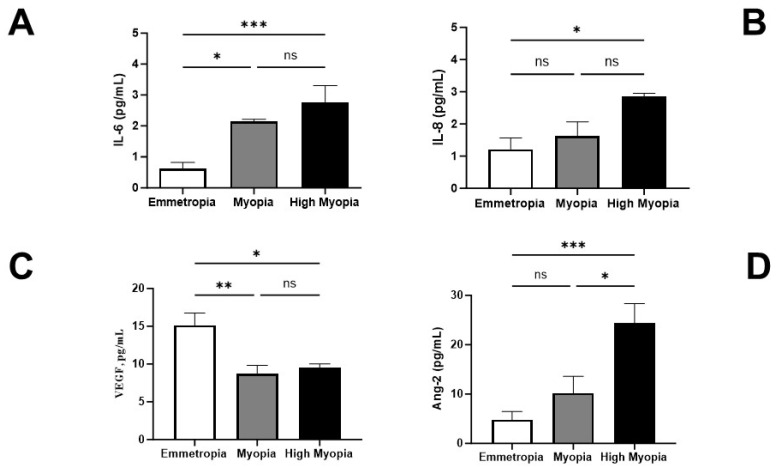
IL-6, IL-8, VEGF-A, and ANG-2 protein levels in AH. Biosamples were collected and categorized according to axial length (AL) elongation (see MM). Microfluidic analysis detected increasing protein levels of IL-6 (**A**), IL-8 (**B**), and ANG2 (**D**) in high myopia and myopia subgroups as compared to emmetropia ones. All changes were AL-elongation-dependent. By contrast, VEGF-A levels (**C**) were reduced in myopia and high myopia with respect to emmetropia, and no difference was detected in myopia groups. IL-6, IL-8, and ANG2 levels were different between high myopia and myopia, but only ANG-2 levels were significant (**D**). Significant levels are shown in the panels (ns, not significant; * *p* ≤ 0.05; ** *p* ≤ 0.005; *** *p* ≤ 0.0005), as calculated using one-way ANOVA followed by a Tukey–Kramer post hoc test (mean ± SEM).

All emmetropic AH displayed detectable amounts of IL-6, IL-8, VEGF-A, and ANG-2, as shown by the white bar graphs in Figure 1A–D. Increased levels of IL-6 and IL-8 proteins were found in AH samples collected from both myopia subgroups, as compared to emmetropic ones (respectively, Figure 1A,B, *p* < 0.05). By contrast, VEGF-A protein levels were decreased in AH samples collected from both myopia subgroups with respect to emmetropic ones (*p* < 0.05; Figure 1C). Of interest, ANG-2 protein levels were increased in AH samples from both myopia subgroups with respect to emmetropic ones (*p* < 0.05; Figure 1D). Significant changes between high myopia and myopia were observed for IL-6, IL-8, and ANG-2 (*p* < 0.05; Figure 1D). No differences in VEGF-A levels were found between high myopia and myopia (*p* > 0.05; Figure 1C).

### 3.2. LECs Express the αSMA-Phenotype (Protein and Transcript) That Correlates with AL Elongation

**Figure 2:**
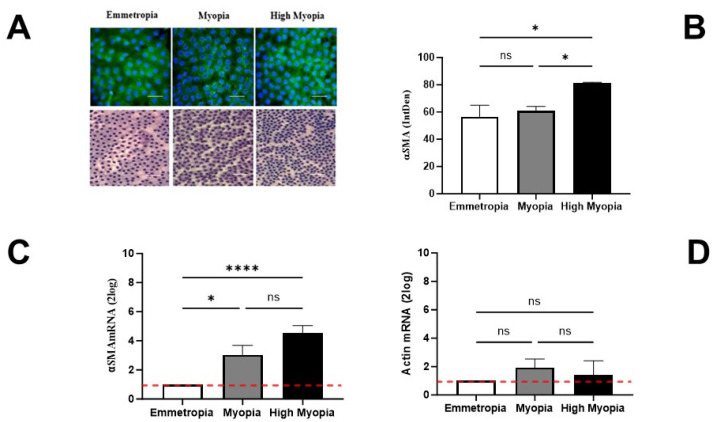
Lens epithelial cells (LECs) display α-smooth muscle actin (α-SMA) phenotypes and synthesize αSMA transcripts. AC/LEC specimens were prefixed at the time of sampling and subjected to specific analysis. (**A**) Representative α-SMA (merge: α-SMA/green and dapi/blue; epifluorescence) and PAS (light microscopy) images are shown. From left to right: upper panels: α-SMA/dapi immunoreactivity (**upper panels**); lower panels: PAS staining (**lower panels**) in emmetropia, myopia, and high myopia (magnifications, ×200; white bar, 100 µm). (**B**) Increased α-SMA immunoreactivity was quantified in highly myopic samples, as compared to myopic and emmetropic ones (* *p* < 0.05). IntDen values were obtained using ImageJ software after the channel split and the background normalization of images. (**C**) Molecular analysis showing increased α-SMAmRNA expressions in highly myopic (**** *p* < 0.0001) and myopic (*p* > 0.05) samples, as compared to emmetropic ones used as the control and referred to as 1 (white box; red-dotted line). (**D**) No changes in actin transcripts were detected between subgroups. (**B**–**D**) Significant levels are shown (data ± SEM) as calculated using one-way ANOVA analysis (ns, not significant; * *p* ≤ 0.05; **** *p* ≤ 0.0001).

AC is an acellular, soft/smooth transparent basement membrane secreted by LECs. When sampled, AC displays a monolayer subcapsular epithelium. This monolayer was analyzed using light and epifluorescence microscopy. An increased expression of α-SMA immunoreactivity was observed in high myopic and myopic samples, as shown in the quadrants of Figure 2A (upper panels, α-SMA/dapi immunofluorescence in emmetropia (left), myopia (middle), and high myopia (right)). This increase was dependent on AL elongation and confirmed via the related integrated density acquisitions (IntDen; Figure 2B). To confirm the quality of IntDen data, PAS staining was used to identify areas with cellularity that were suitable for optic field acquisition and analysis (Figure 2A, lower panels). As shown in Figure 2B, α-SMA IntDen values were significantly increased in high myopia with respect to myopia and emmetropia (* *p* < 0.05), while no difference was detected between myopia and emmetropia (*p* > 0.05). The molecular analysis showed a significant upregulation of α-SMA transcripts in high myopia and myopia RNA extracts with respect to emmetropia (**** *p* < 0.0001; Figure 2C). No significant changes were observed in α-SMA transcripts’ expression between myopia and high myopia (*p* > 0.05; Figure 2C). The analysis of actin transcripts showed no significant changes between subgroups (*p* > 0.05; Figure 2D). As calculated, the α-SMA/Actin ratio was higher in high myopia, supporting the increasing α-SMA immunoreactivity related to increasing AL values (α-SMA/Actin ratio of 1.576 for myopia and 3.032 for high myopia).

### 3.3. LECs Express Transcripts Specific for TGFβR and TGFβ Isoforms

**Figure 3:**
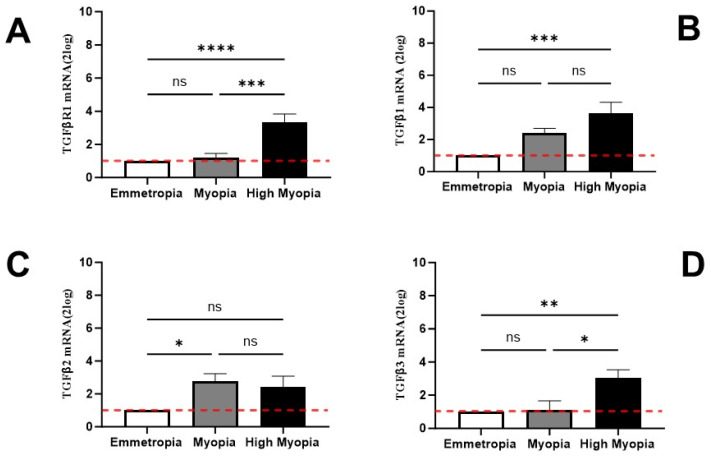
LECs synthesized transcripts specific for TGFβR1 and TGFβ isoforms. LECs were extracted and subjected to real-time RT-PCR. Differences between high myopia and myopia are shown for TGFβ-R (**A**), TGFβ1 (**B**), TGFβ2 (**C**), and TGFβ3 (**D**) and indicated by asterisks, as calculated using one-way ANOVA analysis. Note the significant increase in mRNAs specific for TGFβ-R (**A**) TGFβ1 (**B**) and TGFβ3 (**D**) in high myopia vs. emmetropia. Data are 2log-FC (fold changes, ±SEM), as calculated with respect to emmetropic eyes used as controls and referred to as 1 (white box). Red-dotted lines indicate the level of significance for relative PCR. Significant levels are shown as calculated using one-way ANOVA analysis (ns, not significant; * *p* ≤ 0.05; ** *p* ≤ 0.005; *** *p* ≤ 0.0005; **** *p* ≤ 0.0001).

Microscopical evaluations confirmed the presence of an EMT phenotype in myopic LECs, and this was strictly dependent on AL elongation. To better characterize this EMT phenotype, the expression of the TGFβ profibrogenic family was investigated in the RNA extracts of LECs. As shown, the expression of TGFβ-R1mRNA (Figure 3A) was significantly upregulated in LECs from high myopia compared to myopia and emmetropia (*p* < 0.05), and no difference was observed between myopia and emmetropia (*p* > 0.05). The analysis of TGFβ isoforms revealed that TGFβ1mRNA (Figure 3B) was specifically increased in high myopia with respect to myopia and emmetropia (*p* < 0.05), and no difference was observed between myopia and emmetropia (*p* > 0.05). TGFβ2mRNA (Figure 3C) was slightly increased only in myopia with respect to emmetropia (*p* < 0.05). Finally, TGFβ3mRNA (Figure 3D) was specifically increased in high myopia (*p* < 0.01), and no differences were detected with respect to myopia and emmetropia as they showed comparable expression. The Pearson rho test was carried out between α-SMA transcripts and TGFβ-RI, TGFβ1, TGFβ2, and TGFβ3, highlighting no significant correlations (*p* > 0.05).

### 3.4. LECs might Contribute and Respond to AH Signature

**Figure 4:**
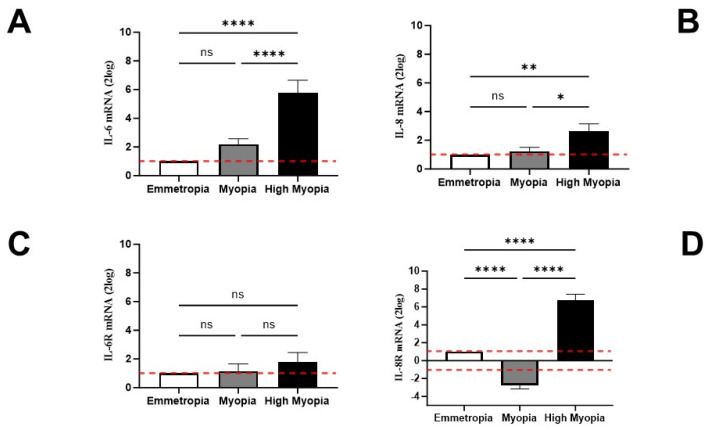
LECs synthesize transcripts specific for IL-6 and IL-8 proteins and related receptors. LECs were analyzed for the specific amplification of IL-6 (**A**) and IL-8 (**B**) transcripts, and their specific IL-6R (**C**) and IL-8R (**D**) receptors were analyzed using relative real-time RT-PCR. A significant transcript expression was observed for IL-6 (**A**) and to a lesser extent for IL-8 (**B**). Of interest, no changes were quantified for IL-6RmRNA (**C**) with respect to IL-8RmRNA transcription that was significantly upregulated in high myopia (**D**). Data are 2log-FC (fold changes, ± SEM), as calculated with respect to emmetropic eyes used as controls and referred to as 1 (white box). Red-dotted lines indicate the level of significance for relative PCR. Significant levels are shown as calculated using one-way ANOVA analysis (*p* value summary: ns, not significant; * *p* ≤ 0.05; ** *p* ≤ 0.005; **** *p* ≤ 0.0001).

To provide information about a possible contribution of LECs in the protein signature of myopic AH, the transcription activities specific for IL-6, IL-8, and VEGF pathways were evaluated with respect to RNA extracts using relative real-time RT-PCR. As shown, an upregulation of IL-6 (*p* < 0.05; Figure 4A) and IL-8 (*p* < 0.05; Figure 4B) transcripts was detected in high myopia in comparison to myopia and emmetropia. The possibility that LECs might respond to the AH signature was verified by analyzing the transcription of specific receptors. Unchanged IL-6RmRNA (*p* > 0.05; Figure 4C) expression was observed between myopic subgroups, and a substantial change in IL-8RmRNA (*p* < 0.0001; Figure 4D) expression was observed in LECs from high myopia to myopia and emmetropia.

**Figure 5:**
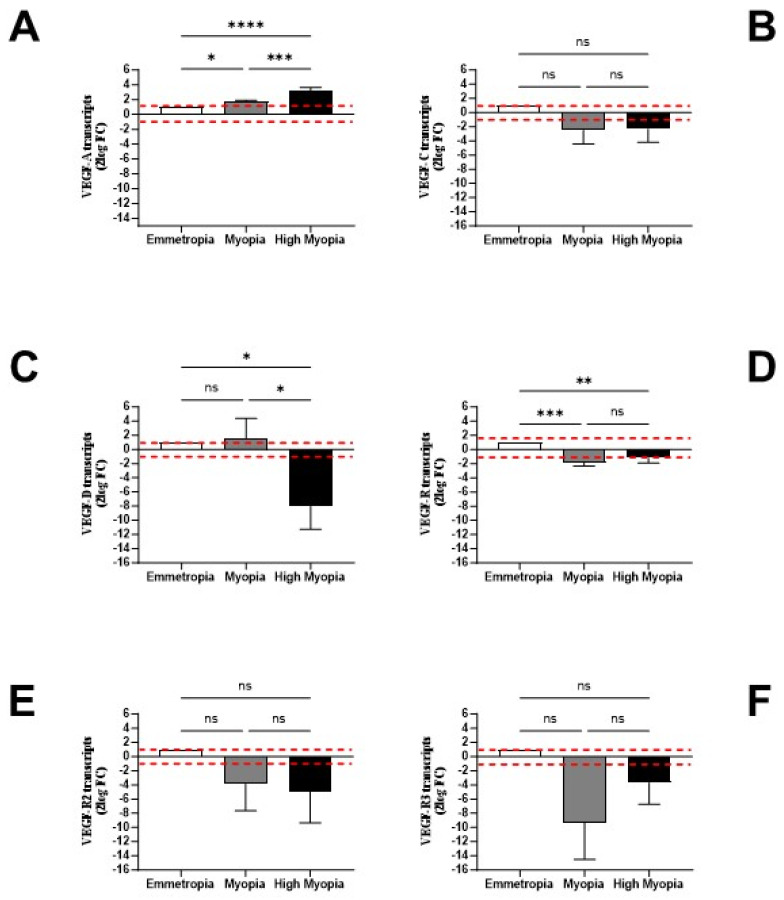
LECs synthesize transcripts specific for VEGF isoform proteins and related receptors. LEC samples were processed, and the specific amplification of VEGF isoform proteins (**A**–**C**) and their specific VEGF-R1 (**D**), VEGF-R2 (**E**), and VEGF-R3 (**F**) receptor transcripts were analyzed using relative real-time RT-PCR. While slight changes were detected for VEGF-R1 (**D**), consistent transcript deregulation was observed for VEGF-R2 (**E**) and particularly VEGF-R3 (**F**) in myopia and high myopia. REST-ANOVA analysis was performed to obtain fold change (FC ± SEM) expression for each subgroup with respect to the emmetropic ones used as a control (herein referred as 1, white box). *p*-values are reported (ns, not significant; * *p* ≤ 0.05; ** *p* ≤ 0.005; *** *p* ≤ 0.0005; **** *p* ≤ 0.0001), as calculated using REST-ANOVA coupled analysis. Red-dotted lines indicate the level of significance for relative PCR.

As shown in Figure 5, the upregulation of VEGF-A (*p* < 0.0001; Figure 5A), unchanged levels for VEGF-C (*p* > 0.05; Figure 5B), and a trend toward a decrease for VEGF-D (*p* < 0.05; Figure 5C) were observed by comparing high myopic groups relative to emmetropic ones. The possibility that LECs might respond in an autocrine/paracrine fashion to VEGF was verified by analyzing the transcription of specific receptors. Except for VEGF-R1 mRNA expression (*p* < 0.05; Figure 5D), no changes were observed for VEGFR2 and VEGF-R3 mRNA (*p* > 0.05; Figure 5E–F).

### 3.5. IL-6R/IL-6 and VEGF-R1/VEGF-A Transcripts Correlate with α-SMA Transcripts

**Figure 6:**
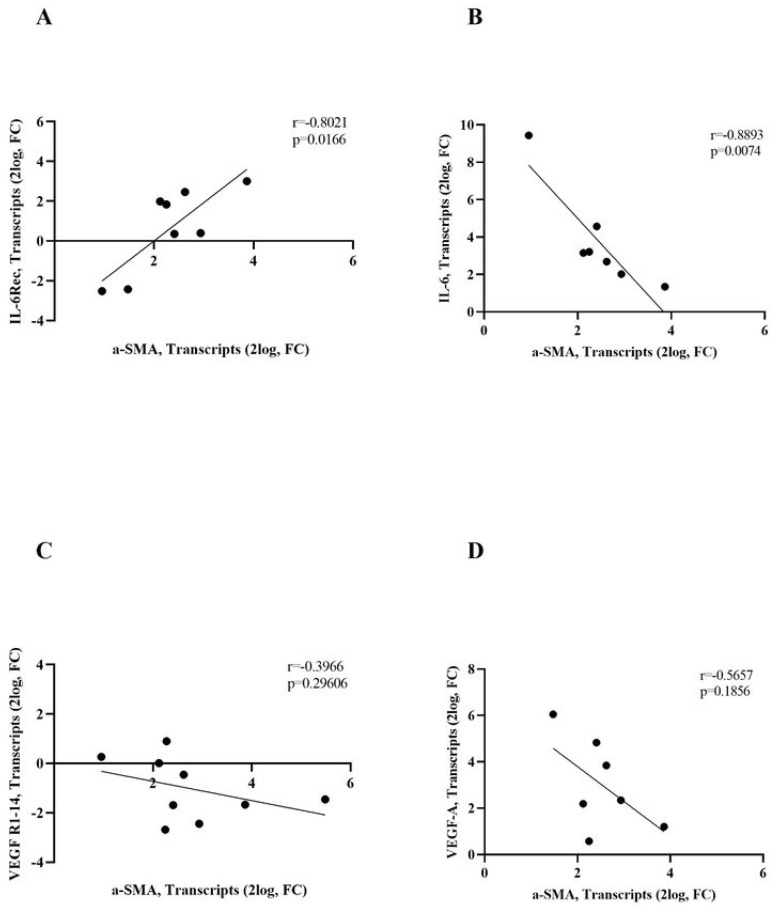
Correlation between IL6 and VEGF pathways and α-SMA phenotype (EMT). Scatterplots representative of IL-6R, IL-6, VEGF-R1-14, and VEGF-A transcripts (2log, FC) plotted against α-SMA (2log, FC). The Pearson rho test showed that IL-6R and IL-6 positively and negatively with the contractile α-SMA transcript expression, respectively. On the contrary, no significant correlation was observed for VEGF-R1 and VEGF-A with respect to α-SMA expression. Correlation data (coefficient and *p* value) are shown in the plot.

The possibility that IL6 and VEGF pathways might be associated with the EMT process was specifically tested. The Pearson rho test analysis showed a direct correlation for IL-6R/α-SMA (Figure 6A; rho = 0.8021, *p* = 0.0166) and an inverse correlation for IL-6/α-SMA (Figure 6B; rho = −0.8893, *p* = 0.0074), as detected in AC. No significant correlation was noted for VEGF-R1/α-SMA (Figure 6C) nor VEGF-A/α-SMA (Figure 6D).

### 3.6. Correlation Analyses for AH Signature and Eye Elongation: Ocular Fluid Circulation

Correlation analyses were also carried out between AL elongation and AH protein print. Precisely, the Pearson rho test was carried out for inflammatory and fibrogenic markers, and it showed a positive and insignificant correlation for IL-6/AL (rho = 0.4907, *p* = 0.0501) and a significant correlation for IL-8/AL (rho = 0.7518, *p* = 0.0048). An inverse significant correlation was detected for VEGF-A/AL (rho= −0.4480, *p* = 0.0476), and of interest, a strong positive correlation was observed for ANG-2/AL (rho = 0.7404, *p* = 0.0004).

## 4. Discussion

The finding of the study i. confirmed a protein profile comprising higher levels of IL-6, IL-8, and ANG-2 and lower levels of VEGF-A in highly myopic AH; ii. showed the presence of the EMT phenotype in myopic AC/LECs; and iii. highlighted LECs’ contribution and response to this AH signature.

Wei et al. reported the presence of IL-6, IL-8, VEGF, ANG-2, and TGFβs in vitreous (VH) in highly myopic patients [25], and more recently, Zhang et al. prospected the therapeutic usefulness of targeting the inflammatory mediators released at the vitreoretinal interface [26]. Therefore, the possibility of crosstalk between the AH protein signature and LECs’ activity and between the AH protein signature and retinal state was hypothesized and herein investigated starting from the few mediators highlighted in Wei and Zhang studies [25,26].

First, the microfluidic analysis of AH confirmed the presence of IL-6, IL-8, VEGF-A, and ANG-2 in myopic samples, highlighting the higher IL-6, IL-8, and ANG-2 levels in high myopic samples. Although this protein expression is not a new finding [9,10,11,22,25,26], our studies extend the literature data by emphasizing IL-6 and IL-8 accumulation with respect to AL elongation. IL-6 and IL-8 are critically involved in the process of inflammation and can also drive some profibrogenic activities and many aspects related to the control of ocular growth, scleral remodeling, and choroidal status [27,28]. Recently, it has been reported that increased inflammation promotes the progression of myopia, whereas decreased inflammation slows the development of myopia, as observed when using intravitreal injections of neutralizing anti-VEGF [29,30,31]. The low ratio of VEGF-A/ANG-2 detected in AH and LECs would suggest reduced angiogenesis and points to the presence of vasculogenesis [32]. This aspect is of great importance as previous studies showed that the simultaneous neutralization of VEGF-A and ANG-2 was able to reduce vascular leakage, immune cell reactivity, and apoptosis in a murine model of aberrant retinal angiogenesis [33]. Studies with neutralizing anti-VEGF antibodies showed low-grade retinal neovascularization in highly myopic subjects [34]. High VEGF levels might imply a conspicuous regulation of ECM, the expression of inflammatory genes in endothelial cells, and corneal neovascularization [14,15]. Thus, the possibility of discriminating a high VEGF-A/ANG-2 ratio in myopic eyes might be of great importance as corneal endothelial cells are directly in touch with the AH as LECs. The herein observed low VEGF-A/ANG-2 and high VEGF-A/VEGF-D ratios deserve further in vitro evaluations.

Second, the analysis of LECs from intact AC samples from high myopic and myopic patients confirmed the presence of an EMT-associated phenotype, as previously reported for cataract LECs [35]. The expression of αSMA in these LECs’ monolayers was increased in an AL-dependent fashion, in line with previous studies on posterior capsule opacity [36]. LECs comprise a simple cuboidal epithelial monolayer located in the anterior portion of the lens between the capsule and fibers [37]. While a healthy lens comprises different regional subsets (non-dividing central ECs, dividing germinative ECs, and fully differentiated internal fiber cells), an insulted lens results in the abnormal growth of fiber cells (myofibroblast-like phenotype); an imbalance of ion/metabolites transport; and the uncontrolled processing of matrix molecules, oxidative mediators; and cell apoptosis (lens opacification) [38]. Herein, EMT differentiation was confirmed due to a specific morphological appearance and the increased expression of TGFβ1, TGFβ3, TGFβ-R1, and αSMA transcripts that are strongly dependent on AL elongation. The higher transcripts’ expression of IL6, IL8, and VEGFA in LECs would suggest that LECs can contribute actively to the myopic AH protein profile. In particular, AH biomarkers (IL6, IL8, VEGFA, and ANG-2) correlated with LECs contractile activity both at the biochemical (α-SMA) and biostrumental (AL elongation) levels. Considering the high levels of IL6, IL8, VEGFA, and ANG-2 in myopic AH, the question was whether changes in myopic AH protein signature might in turn influence LEC activities. The observation of the presence of receptors specific for all these increased biomarkers sustains the hypothesis of an autocrine/paracrine utilization of these mediators by LECs. Of those, IL-6R and VEGF-R1 transcripts seem to be uninfluenced by their specific ligand concentrations. Our comparative studies suggest the hypothesis that highly myopic LECs can produce IL-6, IL-8, and TGFβ1 and particularly show that IL-8, TGFβ1/3, and to a lesser extent IL-6 can be utilized by these LECs in an autocrine/paracrine manner. In particular, the isoforms of TGFβ were differentially expressed. As expected, TGFβ and IL-6 were found positively correlated with the α-SMA contractile phenotype.

A recent study reported the participation of inflammation in the progression of myopia [39]. Scleral remodeling results in AL elongation and thereafter the development of myopia. In previous studies, the elevated levels of tissue remodeling enzymes (MMPs/TIMPs) and soluble inflammatory mediators in AH were associated with an elongated eye axis, suggesting some explanation for the development and progression of myopia [6,7,8]. AL elongation is primarily due to i. the rate of expansion of the scleral shell, which is enriched by extracellular matrix (ECM) connective tissues produced by scleral fibroblasts; and ii. the process of inflammation (NFκB, TGF-βs, MMPs/TIMPs, TNFα, IL-6, and IL-1β), which is most probably produced/released by LECs. Herein, IL-6, IL-8, and ANG-2 correlated positively with AL elongation. Of interest, a negative correlation was observed between VEGF-A and AL elongation [40]. A clear explanation of the low VEGF-A/Ang-2 ratio and particularly the ANG-2-positive and VEGF-A-negative correlations with AL elongation is missing. In vitro studies on cultured LECs might elucidate these aspects using pathological LECs and neutralizing factors (mediators) or control LECs and pathological AH (differentiation studies). Regarding the appearance of the EMT phenotype, the participation of other mediators such as FGF, EGF, NGF, and BDNF, which are known to be increased in AH and able to work as modulators of cell differentiation/contractile activity, cannot be excluded [41,42,43,44,45,46,47]. In previous studies, these growth factors have been reported to be profibrogenic and anti-inflammatory [46,47]. In particular, NGF has been reported to trigger the EMT phenotype and TGFβ and FGF, but the presence of two specific receptors (trkA^NGFR^ and p75^NTR^) allows NGF to mediate a selective response in EMT cells from survival to apoptosis, suggesting additional homeostatic considerations [46,47].

Some limitations can be associated with this observational single-point study with no clinical follow-up. The first limitation might be related to the process of cataractogenesis that might lead to the loss of transparency with respect to the lens due to several factors (age, nutrition, and lifestyle) [48,49,50]. The second limitation is the presence of systemic comorbidities linked to aging (cardiovascular, diabetic, and metabolic diseases) [51,52]. Although these limitations were equally distributed among subgroups (according to inclusion/exclusion criteria), a wider population might provide stronger support for the future usage of the “AH protein signature” as mirroring retinal statuses [52]. However, these findings prompt specific biomarker expressions between inflammatory, vasculogenic, and profibrogenic targets, suggesting the usage of precise neutralization approaches [53,54].

**Figure 7:**
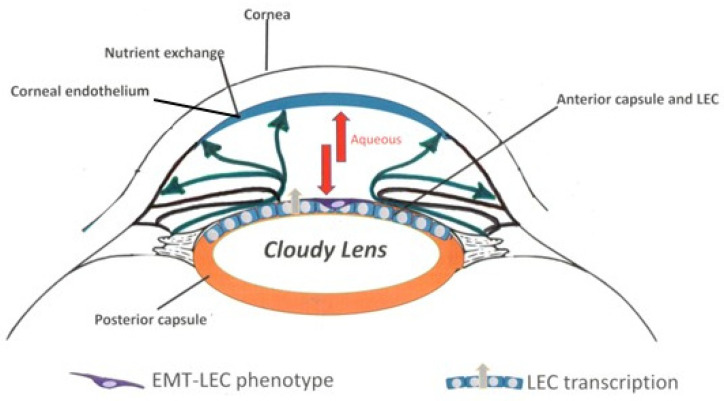
AH circulation and the possible mechanisms behind the myopic AH signature and LEC differentiation. Schematic representation of the anterior chamber (the internal region between cornea and lens). Properly, AH is the result of blood flows entering the ciliary processes (diffusion), the pressure of plasma in the interstitium (ultrafiltration), and the active secretion of mediators by ciliary processes (secretion). AH circulates inside the anterior chamber (dark green lines). In our model, the inflammatory process of cataractogenesis (cloudy lens) and myopia (AL elongation) can influence the entire system, as supported by the presence of a plethora of proinflammatory and profibrogenic mediators. Double red arrows indicate the myopic AH that, in the context of a cloudy lens (cataract), can affect the two major cell monolayers: LECs and corneal endothelial cells. In this environment, LECs might differentiate in response to the AH profile (see a representative contractile EMT-LEC phenotype) or contribute to the AH signature (LEC and EMT-LEC transcription; see gray arrows). In our model, we cannot exclude the possibility that the AH protein signature might also affect corneal endothelial cells. Finally, the space between the cornea and iris, filled by AH (double arrows indicating fluid circulation), can communicate with the posterior chamber via the pupil (see dark green lines), contributing to an enrichment of the vitreal protein signature, as demonstrated in previous studies.

Physiologically, the AH protein signature is the result of i. passive blood flow diffusion and ultrafiltration during ciliary processes and ii. active secretion by the two epithelial layers (secretory cells). Therefore, differences in the composition between plasma and AH are due to the blood–aqueous barrier and are influenced by systemic states. Impairments in the blood–aqueous barrier have been suggested in cases of uveitis and degenerative high myopia. While the blood–aqueous barrier’s breakdown allows the entering of inflammatory cells and soluble mediators during uveitis, the blood–aqueous barrier looks intact in young high myopia with healthy vitreous and fundus [55,56]. This would imply that an active production of mediators might exist inside the anterior chamber, reinforcing the contribution of EMT-LECs. A schematic representation of AH circulation with possible mechanisms occurring inside the anterior chamber is illustrated relative to our results (Figure 7).

Currently, we cannot provide a clinical application of this AH signature in highly myopic patients. Moreover, we cannot exclude the possibility that an inflamed AH might influence the posterior capsule or vitreal composition (retina) or corneal endothelial cells. Considering that excessive AL elongation is often associated with the stretching and thinning of the retina, choroid, and sclera, our data might be utilized for developing alternative approaches with respect to “agents with counteracting properties” and for protecting the vitreoretinal compartment from myopic-linked insults. This aspect is explained by the fact that i. the circulation of ocular fluids between the two chambers cannot be excluded and ii. high myopia is often associated with degenerative changes occurring in the posterior region (posterior staphyloma, choroidal neovascularization, and tractional maculopathy) [52,53,55,56].

## 5. Conclusions

Cataracts often develop as part of the natural aging process and seem to correlate with genetic/epigenetic factors and lifestyle (aging, nutrition, and lifestyle) [57,58,59]. Recent studies suggest that time spent outdoors and more exposure to violet light slow both the onset and progression of myopia in children [60,61]. Unprotected exposure to sunlight (UVA/UVB), such as solar radiation and artificial light, can worsen the natural process of protein breakdown, and highly myopic subjects can develop early cataracts [62,63]. Due to eye elongation, myopic subjects can also develop retinal degeneration (11.4% high myopia), new vessels under the retina (also known as areas of neovascularization), and slight atrophy of the macula [50,51,52]. Under this scenario, our findings suggest that it is possible to provide some predictive tools for alerting clinicians about the status of the vitreo-retinal chamber of highly myopic eyes by coupling biostrumental and biomolecular analyses [64]. Moreover, the knowledge of biomarkers associated with myopic LEC differentiation/contraction might provide countermeasures for severe complications (glaucoma, retinal detachment, choroid retinal atrophy, and macular hole) that are often a result of tissue remodeling and neurodegeneration [65,66]. In conclusion, this study opens up the possibility of an alternative hybrid approach for monitoring myopia progression with an early non-invasive analysis of subclinical parameters [64]. Studies are underway to associate this AH profile with the status of highly myopic retinas using next-generation retinal imaging.

## Figures and Tables

**Table 1:** Study population.

Study Population		Emmetropia	Myopia	High Myopia	*p* Value
Subjects	102	38	32	32	*p* > 0.05
Age	74.87 ± 9.47	78.22 ± 7.43	77.7 ± 7.80	67.71 ± 9.78	*p* > 0.05
Sex F/M	58/44	23/15	13/19	22/10	*p* > 0.05
Axial Length	24.98 ± 2.77	23.04 ± 0.51	24.92 ± 0.41	29.57 ± 0.66	***p* < 0.05**

Clinical and biostrumental examinations were performed at the time of recruitment, and patients were classified according to axial length (AL) elongation values (emmetropia, myopia, and high myopia). Anamnesis included family history and systemic and local comorbidities. Data are mean ± SD. Statistically significant *p* values were obtained according to the one-way ANOVA followed by a Tukey’s HSD post hoc test. *p* value ≤ 0.05 indicates that the difference is statistically significant, as shown in bold. M, Male; F, female; age (years).

## Data Availability

Data are contained within the article or supplementary material.

## Supplementary Materials

The following supporting information can be downloaded at https://www.mdpi.com/article/10.3390/biom13091328/s1. Table S1: Primer sequences, annealing temperature, and amplicon length for relative real-time PCR.
