# Supplementary material for: Biomarker Signature in Aqueous Humor Mirrors Lens Epithelial Cell Activation: New Biomolecular Aspects from Cataractogenic Myopia
Source: Biomolecules. 2023 Aug 29;13(9):1328. doi: 10.3390/biom13091328 (PMC10526747; doi:10.3390/biom13091328)
Supplement: Supplementary file 1 [file biomolecules-13-01328-s001.zip › biomolecules-2449222-supplementary.pdf]

Table S1. Primer sequences, annealing temperature, and amplicon length for relative real-time PCR

PRIMER	*Accession (version)*	*Sequences (For/Rev)*	*bps*
IL6	BC015511	F: 5'-GAC AGC CAC TCA CCT CTT CA-3'<br>R: 5'-CAG TGC CTC TTT GCT GCT TT-3'	125
IL6R	BC132686.1	F: 5'-TTG TTT GTG AGT GGG GTC CT-3'<br>R: 5'-CGA CGC ACA TGG ACA CTA TG-3'	195
IL8	BC013615.1	F: 5'-TCT CTT GGC AGC CTT CCT G-3'<br>R: 5'-TGG GGT GGA AAG GTT TGG-3'	116
IL8R	M68932.1	F: 5'-TGT GAG GAG CTG GAA ACA CA-3'<br>R: 5'-GAT GTT GGT CAT GGG GGT AA-3'	115
VEGF-A	AF022375.1	F: 5'-TGA CAG GGA AGA GGA GGA GA-3'<br>R: 5'-CGG TGT TCC CAA AAC TGG-3'	141
VEGF-C	BC035212.1	F: 5'-GGG AAG GAG TTT GGA G-3'<br>R: 5'-TAC TGG TTT GGG GCC TTG-3'	180
VEGF-D	D89630.1	F: 5'-ATC CCA TCG GTC CAC TAG G-3'<br>R: 5'-GGT ACT CTT CCC CAG CTC ACT-3'	139
VEGFR1	*EU368830.1*	F: 5'-TCG TGT AAG GAG TGG ACC AT-3'<br>R: 5'-TGC CAG CTA CGG TTT CAA G-3'	116
VEGFR2	AF063658.1	F: 5'- CAG ACG GAC AGT GGT ATG GTT-3'<br>R: 5'-GCT TGT CTG GTT TGA GCC TTC-3'	141
VEGFR3	AY233383.1	F: 5'- GAG GTA CAT GCC AAC GAC AC-3'<br>R: 5'-GCT GCT CAA AGT CTC TCA CG-3'	115
ANG-2	BC143902.1	F: 5'-GTG ATC TTG TCT TGG CCG CA-3'<br>R: 5'-AGT GTA GCT GCA GGA CCC AT-3'	101
αSMA	BC017554.2	F: 5'-GAA GGA GAT CAC GGC CCT A-3'<br>R: 5'-ACA TCT GCT GGA AGG TGG AC-3'	125
Actin	J05192.1	F: 5'-AGG CGG TGC TGT CTC TCT AT-3'<br>R: 5'-GGA CAA TCT CAC GCT CAG CA-3'	219
TGFβ1	BC017288	F: 5'-GAG ATG AGG GTT TCC ACG AG-3'<br>R: 5'-GCG CCG AGA TGT AGT TAT CC-3'	120
TGFβ2	BC096235.4	F: 5'-GGA GTA CTA CGC CAA GGA GGT-3'<br>R: 5'-TAG ACG GCA CGA AGG TAC AG-3'	121
TGFβ3	BC014690	F: 5'-GAG CCC CTT TGA CCA TCT TGT A-3'<br>R: 5'-GCA GTT TTC CCT CCT CTG TG-3'	121
TGFβRI	BC071181.1	F: 5'-CAG CTC TGG TTG GTG TCA GA-3'<br>R: 5'-ATG TGA AGA TGG GCA AGA CC-3'	131
H3	NM005324.4	F: 5'-ACC TGG CTA CGG TGA TGA GT-3'<br>R: 5'-GAG TGC TCA AGC CAA GTG TG-3'	174
GAPDH	BC013310.2	F: 5'-AAC AGC GAC ACC CAC TCC T-3'<br>R: 5'-GGT CCA GGG GTC TTA CTC CTT-3'	162

Specific amplifications were tested by verifying the single curve specific for each amplicon. Hot-start SYBRgreen Hydra mix was activated by a pre-hold (5min at 50°C) and pre-incubation for 15min at 95°C. Each of the 39 amplification cycles consisted of a 30sec/94°C (denaturation), followed by a specific annealing step 58–60°C and a 30sec/72°C (extension). Annealing was set at appropriate temperature (Tm – 5°C) verified for specificity by grading. Melting curve was registered from 56.0°C to 94.1°C; 0.3°C; hold for 00:00:01 between reads.
